# Potential role of AKT/mTOR signalling proteins in hairy cell leukaemia: association with BRAF/ERK activation and clinical outcome

**DOI:** 10.1038/srep21252

**Published:** 2016-02-19

**Authors:** Eleftheria Lakiotaki, Georgia Levidou, Maria K. Angelopoulou, Christos Adamopoulos, Gerassimos Pangalis, George Rassidakis, Theodoros Vassilakopoulos, Gabriella Gainaru, Pagona Flevari, Sotirios Sachanas, Angelica A. Saetta, Athanasia Sepsa, Maria Moschogiannis, Christina Kalpadakis, Nikolaos Tsesmetzis, Vassilios Milionis, Ilenia Chatziandreou, Irene Thymara, Panayiotis Panayiotidis, Maria Dimopoulou, Eleni Plata, Konstantinos Konstantopoulos, Efstratios Patsouris, Christina Piperi, Penelope Korkolopoulou

**Affiliations:** 1 Department of Pathology, University of Athens, Medical School, Greece; 2 Department of Haematology and Bone Marrow Transplantation, University of Athens, Medical School, Greece; 3 Department of Biological Chemistry, University of Athens, Medical School, Greece; 4 Department of Haematology, Athens Medical Centre, Psychikon Branch, Greece; 5 Department of Oncology-Pathology, Cancer Centrum Karolinska, Karolinska Institutet, Stockholm, Sweden; 6 Department of Haematology, University of Crete, Heraklion, Greece; 7 1st Department of Propaedeutic Internal Medicine, University of Athens, Medical School, Greece

## Abstract

The potential role of AKT/mTOR signalling proteins and its association with the Raf-MEK-ERK pathway was investigated in hairy cell leukaemia (HCL). BRAFV600E expression and activated forms of AKT, mTOR, ERK1/2, p70S6k and 4E-BP1 were immunohistochemically assessed in 77 BM biopsies of HCL patients and correlated with clinicopathological and BM microvascular characteristics, as well as with c-Caspase-3 levels in hairy cells. Additionally, we tested rapamycin treatment response of BONNA-12 wild-type cells or transfected with *BRAFV600E*. Most HCL cases expressed p-p70S6K and p-4E-BP1 but not p-mTOR, being accompanied by p-ERK1/2 and p-AKT. AKT/mTOR activation was evident in BONNA-12 cells irrespective of the presence of *BRAFV600E* mutation and was implicated in cell proliferation enhancement. In multivariate analysis p-AKT/p-mTOR/p-4E-BP1 overexpression was an adverse prognostic factor for time to next treatment conferring earlier relapse. When p-AKT, p-mTOR and p-4E-BP1 were examined separately only p-4E-BP1 remained significant. Our findings indicate that in HCL, critical proteins up- and downstream of mTOR are activated. Moreover, the strong associations with Raf-MEK-ERK signalling imply a possible biologic interaction between these pathways. Most importantly, expression of p-4E-BP1 alone or combined with p-AKT and p-mTOR is of prognostic value in patients with HCL.

Establishment of *BRAFV600E* mutation as the molecular hallmark for hairy cell leukaemia (HCL) was first reported by Tiacci *et al.*^1^ and subsequently confirmed by others^2^,^3^,^4^,^5^. Remarkably, neither HCL variant (HCLv) nor HCL expressing IgHV4–34 immunoglobulin rearrangement nor any other type of peripheral small B cell lymphoma reportedly carried this molecular abnormality^2^,^6^,^7^. Several strategies have been used for the detection of *BRAF* mutation in bone marrow (BM) samples by different investigators, including sequencing, allele-specific quantitative PCR^8^,^9^,^10^, HRMA^11^ and pyrosequencing assays^12^, or immunohistochemistry using a newly developed mutant-specific diagnostic antibody^13^,^14^. Besides being a diagnostic tool, *BRAFV600E* identification may revolutionize current therapeutic considerations, justifying the use of BRAF and MEK inhibitors for the treatment of refractory HCL^15^.

BRAF belongs to the RAF serine/threonine protein kinase family and in normal cells serves as a mitotic signal transporter in the Ras/Raf/mitogen-extracellular signal-regulated kinase 1/2 (MEK1/2)/extracellular signal regulated kinase 1/2 (ERK1/2) (MAPK) pathway, playing a pivotal role in regulating embryogenesis, cell proliferation, differentiation and survival^16^. It represents the most frequently mutated protein kinase gene^17^. The most common *BRAF* mutation (*V600E*) occurs at position 1799, identified as T>A transversion^6^. *V600E* substitution resides within the activation loop of *BRAF*, thereby leading to its constitutive activation and phosphorylation of MEK1 and MEK2, which in turn phosphorylate and activate the effector kinases ERK1 and ERK2^9^,^18^,^19^,^20^. ERKs target numerous substrates, such as protein kinases, transcription factors and cytoskeletal or nuclear proteins. Moreover, they are able to affect protein functions either by phosphorylating proteins in the cytoplasm or by translocating into the nucleus where they activate transcription factors regulating proliferation and cell survival associated genes^21^. The immunohistochemical visualization of phosphorylated (p)-ERK may be advantageous in some respects to routine PCR testing for *BRAF* mutations yielding information regarding the activation of MAPK pathway for reasons other than *BRAF* mutation^22^.

The MAPK pathway elaborates several interconnections with AKT (v-akt murine thymoma viral oncogene homologue 1)/mTOR (mammalian target of rapamycin) signalling pathway^23^,^24^. This is a key intracellular signalling cascade strategically positioned at the convergence point of several oncogenic pathways which acts as a regulator of cell growth, differentiation and migration^25^,^26^. Once activated, AKT kinase phosphorylates several substrates including mTOR kinase. mTOR is a serine/threonine kinase which controls protein synthesis, angiogenesis and cell proliferation and exists in two functionally distinct complexes within the cell, the rapamycin-sensitive mTORC1 and the rapamycin-insensitive mTORC2 complexes. mTOR can be activated by AKT via abrogation of the inhibitory function of the tuberous sclerosis complex (TSC)^27^. Following its activation, mTORC1 phosphorylates its downstream targets p70S6K serine/threonine kinase and 4E-BP1 initiation binding protein. Upon phosphorylation, the latter releases elF4E in order to interact with elF4G. This enables the formation of the elF4F complex, which is responsible for the increased translation of 5′ terminal oligopyrimidine tract mRNAs^25^,^28^.

Activation of Raf-MEK-ERK pathway in HCL is well established^9^. However, the potential role of the AKT/mTOR signalling and its possible associations with the Raf-MEK-ERK pathway have not been studied thus far. In the present study, we hypothesized that the AKT/mTOR signalling is activated in HCL and might be linked to the Raf-MEK-ERK pathway. Therefore, the activation status of critical components of these pathways was assessed by immunohistochemistry in 77 BM biopsies of previously untreated patients with HCL and was confirmed by Western blot analysis in a limited number of BM aspirates. The results were correlated with clinicopathological and BM microvascular characteristics, as well as the apoptotic potential of hairy cells assessed by Caspase-3 activation (c-Caspase-3). Although the reliability of the commercially available human HCL cell lines has been questioned^29^,^30^, the BONNA-12 cell line carrying the wild-type *BRAF* gene was stably transfected with a *BRAFV600E* mutant in order to investigate activation of AKT/mTOR pathway, response to rapamycin and cell proliferation effects.

## Results

### Activation of the AKT/mTOR signalling proteins and c-Caspase-3 expression in HCL

**Figure 1:**
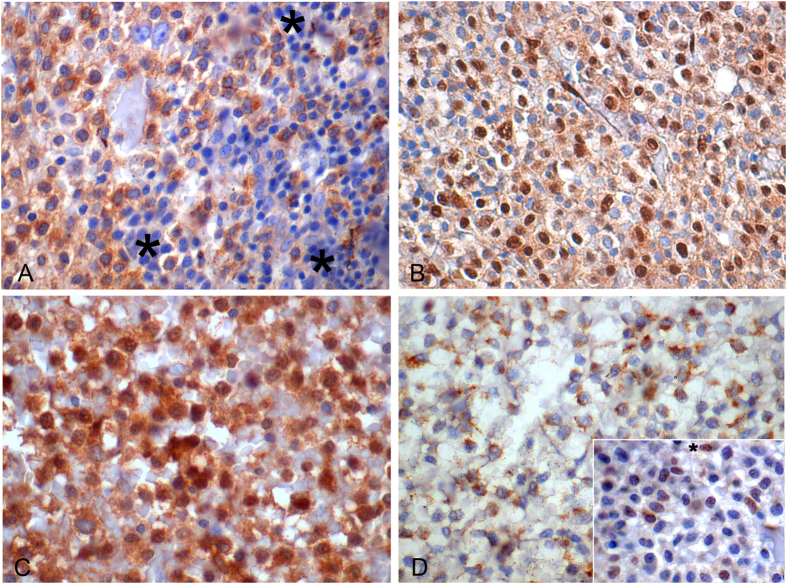
p-mTOR, p-p70S6K, p-4E-BP1 and p-AKT expression in HCL (X600). (**A**) Cytoplasmic p-mTOR immunoreactivity in hairy cells. Note the negative precursor erythroid cells marked with black stars (*). (**B**) Nuclear and cytoplasmic p-p70S6K immunoreactivity in hairy cells. (**C**) Nuclear and cytoplasmic p-4E-BP1 immunoreactivity in hairy cells. (**D**) Punctate cytoplasmic p-AKT immunoreactivity in hairy cells. Note the presence of scattered positive nuclei (*) in another case in inset.

**Figure 2:**
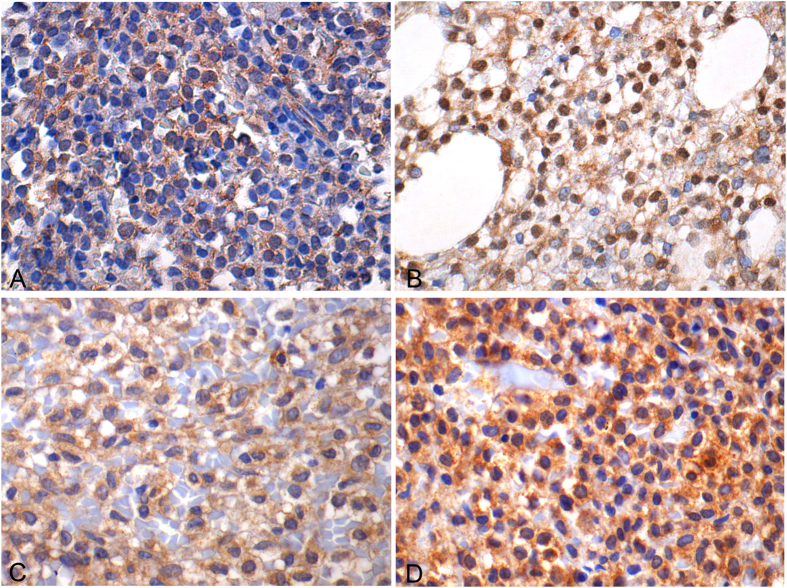
p-ERK1/2 and BRAFV600E expression in HCL (X600). (**A,B**) p-ERK1/2 expression in HCL. In case (**A**) cytoplasmic positivity is identified, whereas case (**B**) shows a diffuse nuclear expression pattern accompanied by some cytoplasmic positivity. (**C,D**) BRAF V600E faint (**C**) and intense (**D**) cytoplasmic expression in HCL.

The rate of expression and coexpression of the various proteins by hairy cells are shown in Table 1. Cytoplasmic p-mTOR immunopositivity was present in only ~13% of the cases (Fig. 1A), whereas cytoplasmic p-p70S6K and p-4E-BP1 expression (Fig. 1B,C) was seen in the vast majority of cases, with nuclear immunoreactivity of both molecules being distinctly uncommon. All p-mTOR immunopositive cases coexpressed p-p70S6K and p-4E-BP1. All p-p70S6K positive/p-mTOR negative cases expressed p-ERK1/2, BRAFV600E and p-AKT. Moreover, all p-4E-BP1 positive/p-mTOR negative cases were positive for p-ERK1/2 and all but one for p-AKT, indicating that both p70S6K and 4E-BP1 can be activated by the BRAF/ERK pathway and p-AKT in a large subset of HCL cases. None of our HCL cases was negative for all three components of the mTOR cascade, suggesting that mTOR signalling is activated in HCL irrespective of the upstream mechanism (AKT or BRAF/ERK).

p-AKT immunoreactivity was cytoplasmic (Fig. 1D), accompanied by some nuclear positivity in a few cases (Fig. 1D inset). The ten cases that were positive for the three mTOR pathway components were also positive for p-AKT. Cytoplasmic expression of p-ERK1/2 was observed in all cases, with only a minority displaying also nuclear expression (Fig. 2A,B). Endothelial cells were positive and served as internal controls.

Expression of the BRAFV600E mutant protein was identified in all cases with a median H-score of 100 (range 5–300) (Fig. 2C,D). In the majority (53/77, 68.8%) of the examined cases, all neoplastic cells expressed BRAFV600E. Of the remaining cases, only 3 displayed immunoreactivity in <20% of the neoplastic population. BRAFV600E staining intensity was distributed as follows: faint positivity in 8/77 (10.38%, Fig. 2C), moderate positivity 55/77 (71.42%) and intense positivity in 14/77 (18.18%, Fig. 2D). Intense staining was also observed in BM mast cells.

c-Caspase-3 was analysed to investigate the potential involvement of the examined proteins in the apoptotic process of hairy cells. c-Caspase-3 expression was observed in all examined cases being restricted to hairy cells and showing a fine granular cytoplasmic staining pattern and a low median H-score (40) as expected.

**Figure 3:**
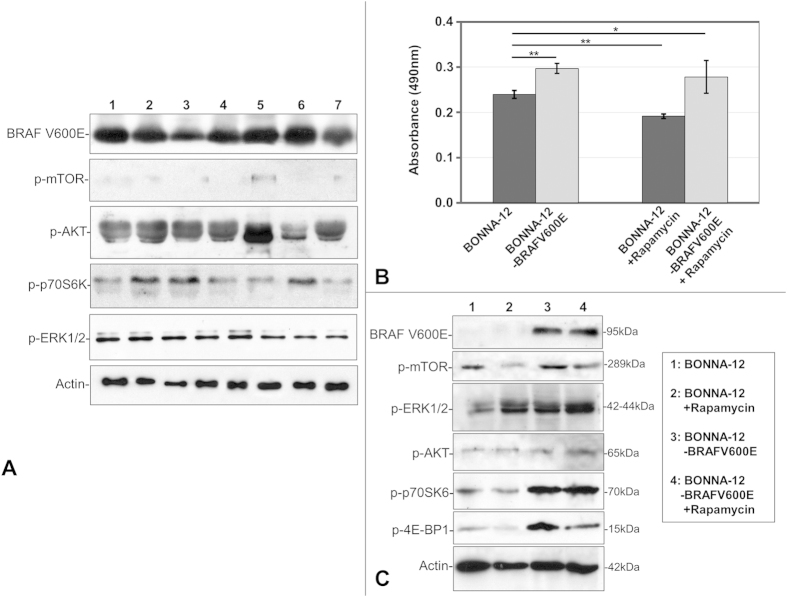
Western immunoblot of BRAF V600E, p-ERK1/2, AKT/mTOR and BONNA-12 proliferation assay. (**A**) Western immunoblot indicating BRAFV600E, AKT/mTOR pathway components and p-ERK1/2 protein expression in 7 HCL tumour samples. All experiments have been performed at least three times and representative results of one experiment are shown. (**B**) Cell proliferation assay of BONNA-12 cells carrying the wild type or *V600E* mutant *BRAF* gene before and after rapamycin treatment (500 nM) for 48 hrs. (**C**) Western immunoblot indicating BRAF V600E, p-ERK1/2, AKT /mTOR cascade protein expression in BONNA-12 cells carrying the wild-type or *V600E* mutant *BRAF* gene before and after rapamycin treatment.

Immunohistochemical data were validated by Western blot analysis performed in BM aspirates from 7 HCL patients. Protein expression of AKT/m-TOR pathway components, BRAFV600E and p-ERK1/2 was similar to the respective immunohistochemical expression pattern (Fig. 3A).

### Associations among AKT/mTOR pathway components, BRAFV600E, p-ERK1/2 and c-Caspase-3 expression, as well as with microvascular characteristics and clinicopathological features

The correlations among the various proteins are shown in Table 1. BRAFV600E correlated positively with nuclear p-p70S6K, cytoplasmic p-4E-BP1 and nuclear/cytoplasmic p-ERK1/2. Cytoplasmic p-ERK1/2 correlated with p-mTOR, cytoplasmic p-p70S6K and cytoplasmic p-4E-BP1. p-mTOR correlated with its upstream AKT and its downstream cytoplasmic p-4E-BP1 and p-p70S6K (positively with cytoplasmic H-score and negatively with nuclear H-score). Finally, p-AKT correlated with nuclear (but not cytoplasmic) p-p70S6K. Nuclear p-ERK1/2 expression was positively correlated with shape factor (Supplementary Information Table S1). The remaining examined molecules were not correlated with any of the microvascular characteristics. c-Caspase-3 H-score was positively correlated with cytoplasmic p-p70S6K H-score, but was unrelated to the remaining molecules (Table 1).

The associations between the examined proteins and the clinicopathological as well as microvascular characteristics are shown in Supplementary Information Table S1. Cytoplasmic p-ERK1/2 H-score was inversely correlated with haemoglobin levels and absolute platelet counts. p-AKT H-score was inversely associated with the presence of splenomegaly, although this was of marginal significance, and positively with absolute platelet counts. Nuclear p-p70S6K H-score was negatively associated with the absolute number of circulating hairy cells. BRAFV600E expression (H-score) was negatively correlated with the absolute number of platelets and the absolute number of neutrophils.

### Expression of AKT/mTOR pathway components, BRAFV600E and p-ERK1/2 in other small B-cell malignancies

Neither p-ERK1/2 (cytoplasmic/nuclear) nor BRAFV600E expression was present in normal haematopoietic BM cells or in any case of MCL, MZL, CLL or HCLv tested.

p-AKT, p-mTOR, p-4E-BP1 and p-p70S6K were expressed by a small number of normal haematopoietic cells. p-AKT was faintly positive in 11/16 cases of small B cell lymphomas whereas p-mTOR along with its two effectors were rather strongly expressed by the majority of cases (15/16 for p-mTOR, 12/16 for p-4E-BP1 and 16/16 for p-p70S6K). In particular, p-AKT and p-mTOR highlighted the proliferation centres in CLL. Details regarding the expression of these molecules in control BMs and the various types of small B cell lymphomas are summarized in Supplementary Information Tables S2 and S3*, whereas representative figures are provided in* Supplementary Information Figures S1 and S2.

### Effect of BRAFV600E mutation in BONNA-12 cell proliferation, p-ERK1/2 and AKT/mTOR components

BONNA-12 cells stably transfected with the *BRAFV600E* mutant gene showed elevated proliferation compared to wild-type *BRAF* BONNA-12 cells based on MTS assay. Rapamycin treatment for 48 hours reduced proliferation of cells carrying the wild-type as well as the mutant *BRAF* (Fig. 3B). However, this reduction was more pronounced in the former cells indicating that *BRAFV600E* might confer resistance to rapamycin treatment in this *in vitro* system.

Western blot analysis of both wild-type *BRAF* cells and *BRAFV600E* transfected cells revealed higher p-ERK1/2 and p-mTOR, p-p70S6K and p-4E-BP1 levels in the *BRAF-*mutated cells (Fig. 3C). Rapamycin treatment of both cell lines caused a reduction of only p-mTOR and p-4E-BP1 levels (Fig. 3C).

### BRAF mutational analysis

**Figure 4:**
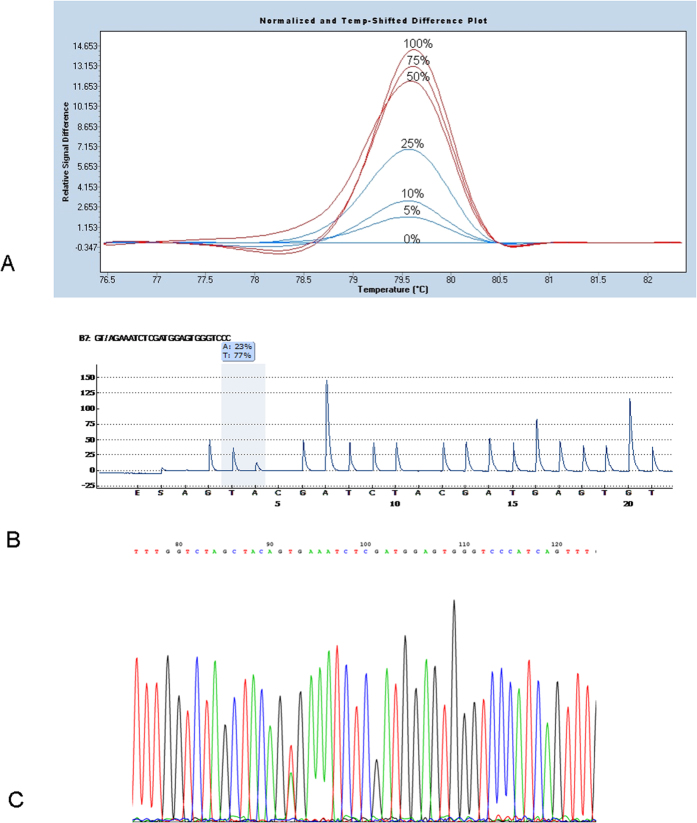
High resolution melting, Sequencing and Pyrosequencing of *BRAF.* (**A**) Dilution series of HT29 DNA which harbours an heterozygous BRAF mutation (p.V600E) analysed by High Resolution Melting. The sensitivity of the assay was defined between 5–10%. (**B,C**) Pyrosequencing and sequencing results for the *BRAF* gene exon 15 respectively showing a substitution T>A at nucleotide 1799 (p.V600E).

Molecular analysis was successful in all 35 cases analysed by HRMA. The detection sensitivity of the technique was defined between 5–10%, as assessed by dilution series of HT29 DNA (Fig. 4). Due to the fact that the samples displayed a variation in the percentage of HRM positivity, *BRAF* mutations were identified by Sanger sequencing and/or pyrosequencing, as appropriate. *BRAF* mutations were observed in 30/35 (85.71%) of the examined samples identified as the common T>A transversion at nucleotide 1799 (c.1799T>A) leading to a valine to glutamic acid substitution at codon 600 (p.V600E) [Mutation Id: COSM476, COSMIC database].

### Survival analysis

**Figure 5:**
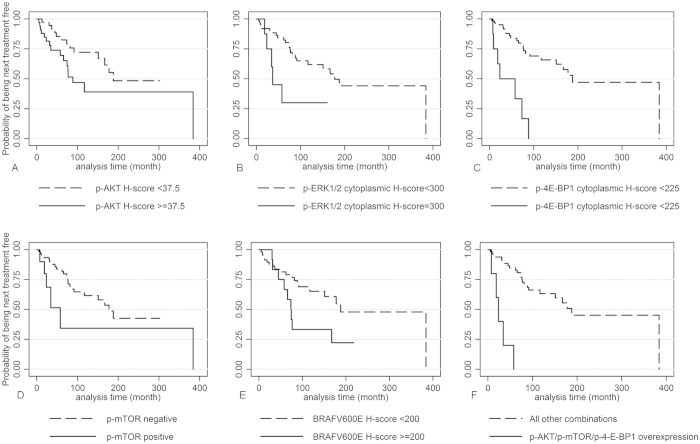
Kaplan Meier survival curves for TNT. Kaplan Meier survival curves for time to next treatment (TNT) according to (**A**): p-AKT H-score (**B**): p-ERK1/2 H-score (**C**): cytoplasmic p-4E-BP1 H-score (**D**): p-mTOR H-score (**E**): BRAF V600E H-score (**F**): concurrent p-AKT/p-mTOR/p-4E-BP1 overexpression.

Information about patient survival and treatment is summarized in Supplementary Information Table S4. In univariate analysis (Table 2) increased BM infiltration, decreased haemoglobin levels, younger age along with increased p-AKT (Fig. 5A), cytoplasmic p-ERK1/2 (Fig. 5B), cytoplasmic p-4E-BP1 (Fig. 5C), p-mTOR (Fig. 5D) expression and BRAFV600E (Fig. 5E) H-score were correlated with shorter time to next treatment (TNT). Moreover, the presence of simultaneous overexpression of p-AKT/cytoplasmic p-mTOR/cytoplasmic p-4E-BP1 was associated with a shorter TNT, indicating earlier relapse (Fig. 5F). The coexpression of these three molecules remained significant in multivariate analysis along with the degree of BM infiltration by hairy cells (*HR* = 69.7, *P* = 0.001, Table 3, ***model A***). When p-AKT, p-mTOR and p-4E-BP1 were entered separately in Cox’s model, only the latter emerged as an independent predictor of adverse prognosis (*HR* = 8.2, *P* = 0.01, Table 3, ***model B***), along with the degree of BM involvement by hairy cells and the type of initial treatment.

## Discussion

The present investigation adds to the existing literature in HCL in various aspects. The activation of AKT and the two mTOR critical downstream targets (p-4E-BP1 and p-p70S6K) is documented for the first time. These molecules appear to elaborate several interconnections with the BRAF/ERK pathway and yield important information with regard to relapse. Our findings are derived from a BM immunohistochemical analysis on 77 homogeneously treated patients followed-up for a median of 10 years and are corroborated by Western blot analysis in a limited number of BM aspirates. Forced expression of *V600E* mutant in BONNA-12 cells verified the contribution of BRAF/ERK to activation of mTOR signalling molecules. Finally, we identified the differences between HCL and other small B cell lymphomas with regard to the expression of BRAF/ERK and AKT/mTOR components.

The vast majority (>90%) of HCL cases displayed cytoplasmic p-AKT, p-p70S6K and p-4E-BP1 immunoreactivity in a significant percentage of the neoplastic population in the absence of p-mTOR denoting that phosphorylation of p70S6K and 4E-BP1 is largely mediated by BRAF/ERK pathway or p-AKT. Of note, p-mTOR positively correlated upstream with p-AKT and downstream with p-p70S6K and p-4E-BP1 indicative of the full activation of mTOR cascade in a small subset of HCL cases. The much higher prevalence of p-AKT compared to p-mTOR positivity points towards a variety of functions of the former which directly phosphorylates many other targets besides mTOR^31^ such as cytoplasmic p-p70S6K via TSC2^25^. Another plausible mechanism responsible for the universal activation of p70S6K as well as of 4E-BP1 in HCL by-passing mTOR may be their phosphorylation directly by MAP kinases^32^,^33^,^34^,^35^ fitting entirely with the correlation between cytoplasmic p-ERK and cytoplasmic p-p70S6K or p-4E-BP1 elicited in our series. Despite the correlation of p-mTOR with both downstream targets, rapamycin treatment caused a decrease of only p-4E-BP1 on BONNA-12 cells, either wild type or *BRAFV600E* transfected. Intriguingly, only nuclear p-p70S6K displayed inverse correlations with p-mTOR or p-AKT and with the absolute number of circulating hairy cells, whereas only cytoplasmic p-p70S6K was associated with c-Caspase-3 levels, consistent with the notion that p-p70S6K may evoke differential effects according to its localization^36^. Notably, in contrast to HCL, all other small B cell lymphomas tested expressed p-mTOR simultaneously with p-AKT, p-p70S6K and p-4E-BP1.

This is the first study exploring the clinical significance of activated AKT/mTOR pathway in HCL. p-AKT, p-mTOR and p-4E-BP1 expression were all associated with a shorter TNT in univariate analysis, either on their own or in combination, along with younger patient age, anaemia, elevated percentage of BM infiltration by hairy cells. This prognostic effect remained in multivariate analysis. Among p-AKT, p-mTOR and p-4E-BP1, only the latter emerged as the independent prognosticator. It should be emphasized that p-AKT expression exerted an adverse prognostic effect, despite its association with smaller spleen size and normal platelet count. Notably, p-p70S6K expression was not informative in this regard.

Of the investigated molecules, only cytoplasmic p-p70S6K positively correlated with c-Caspase-3 expression by hairy cells advocating that AKT/mTOR or BRAF/ERK signalling does not profoundly affect apoptosis. On the contrary, our findings support the involvement of this signalling cascade in the regulation of proliferation since BONNA-12 *BRAFV600E* transfected cells were less sensitive to rapamycin treatment. However, a serious limitation for *in vitro* studies in HCL is that, according to recent literature^29^,^30^; BONNA-12 and all the commercially available cell lines do not represent reliable *in vitro* study models for HCL. Arguably, our *BRAFV600E* transfected cell line could be a model more closely simulating primary HCL cells, as it harbours the same oncogenic mutation.

In the present study, we confirmed by immunohistochemistry (clone VE1) the detection of *BRAFV600E* mutation, which reportedly allows visualization of the respective mutant protein with high sensitivity and specificity^13^,^14^,^37^,^38^, being particularly useful for decalcified BM biopsies^10^,^39^ and the detection of minimal residual disease^14^. As expected^13^,^14^, all our HCL cases were deemed variably positive for BRAFV600E immunohistochemically. Furthermore, as in the study by Andrulis *et al.*^13^, we noted that the variation in VE1 immunoreactivity is unrelated to the electropherogram heights of the sequencing analysis. Our finding of 5 HRMA-negative cases, which nevertheless stained positive by immunohistochemistry, can be attributed to the poor quality of the extracted DNA, as stated by other groups^10^.

p-ERK1/2 expression was documented in all cases, being more often cytoplasmic (100%) than nuclear (23,4%). This dual localization of p-ERK conforms to its function, as it can target both nuclear and cytosolic proteins. Our findings are in broad agreement with those of Warden *et al.*^22^ who, however, reported a nuclear, rather than a cytoplasmic signal in their HCL cases, by using a different antibody which recognizes another phosphorylation site (Tyr214). The coexpression and significant positive correlation of BRAFV600E and p-ERK1/2 H-scores attests to p-ERK serving as a downstream effector of the MAPK pathway, which is constitutively activated due to *BRAF* mutation. This was further confirmed by our study in BONNA-12 cells carrying the *BRAFV600E* mutant gene which exhibited higher p-ERK1/2, but also p-mTOR, p-p70S6K and p-4E-BP1 levels compared to wild-type cells. Importantly, p-ERK1/2 and BRAFV600E expression was not observed in any other small B cell malignancy examined in our study, confirming literature data^14^ and supporting the use of these molecules as diagnostic tools. Arguably, since the fraction of p-ERK1/2 expressing hairy cells was very high, MAPK activation resulting from BRAFV600E mutation is an early and important event in HCL tumourigenesis^22^. Nuclear p-ERK1/2 expression, although seen in a minority of cases, increased in parallel with the shape factor of BM microvessels, which translates to the prevalence of rounder vessel sections indicative of increased intraluminal pressure and dysfunctional microvascular network^40^.

As far as clinicopathological correlations of the above molecules are concerned, elevated BRAFV600E and cytoplasmic p-ERK1/2 H-score were inversely related to haemoglobin levels and absolute number of platelets, implying a role for these proteins in the development of cytopenias in HCL and adversely impacted on TNT in univariate analysis, in harmony with previously reported data in other tumours^17^,^41^,^42^.

In conclusion, we provide novel evidence about the phosphorylation of mTOR downstream targets in the vast majority of HCL cases largely due to independent fuelling by BRAF/ERK and p-AKT. These findings are verified by mechanistic experiments in BONNA-12 cells, in which forced expression of *BRAFV600E* mutant resulted in increased levels of p-ERK1/2, p-mTOR, p-p70S6K and p-4E-BP1. Importantly, p-4E-BP1 on its own or combined with p-AKT and p-mTOR is brought forward as an independent predictor of earlier relapse. Finally, the universal activation of AKT may provide a rationale for new therapeutic considerations in HCL including AKT inhibitors as an adjunct to BRAF and MEK inhibitors^5^.

## Materials and Methods

### Patient characteristics and selection criteria

The patient study group included 77 patients diagnosed with classical HCL between 1980 and 2012 who were treated and followed-up in a single institution. All participants provided written informed consent. The study was approved by the Athens Medical School Ethics Committee (number of study: 5694) and was conducted in accordance with the approved guidelines. All cases were reviewed by an experienced haematopathologist (PK) using the diagnostic criteria established in the 2008 WHO classification^43^. The inclusion criteria were the characteristic cytomorphological features and the expected immunophenotype (coexpression of CD11c, CD25, and CD103 on flow cytometry and positivity for annexinA1 by immunohistochemistry. HCLv cases were excluded from the present investigation. The clinicopathological characteristics and treatment information of the patients appear in Supplementary Information Table S4. Control BMs from 5 cases with no evidence of marrow disease performed as part of staging procedure for Hodgkin lymphoma or plasma cell myeloma were also stained with the primary antibodies listed in Supplementary Information Table S5. Moreover, 5 cases of mantle cell lymphoma (MCL), 5 cases of marginal zone lymphoma (MZL), 5 cases of chronic lymphocytic leukaemia (CLL) and one case of HCLv, were also immunostained with the same antibodies.

### Immunohistochemical analysis

Immunohistochemistry was performed on paraffin-embedded 4 μm-thick sections of formalin-fixed and decalcified (3–5% HCl) BM tissue using the two-step peroxidase conjugated polymer technique (DAKO Envision kit, DAKO, Carpinteria, CA) with the primary antibodies listed in Supplementary Information Table S5. Moreover, information regarding the microvascular characteristics was available in 36 cases from our previous report^44^.

Immunohistochemical light microscopic evaluation was performed by two independent pathologists (PK, EL), without knowledge of the clinical information. Intraobserver variability was <5%, whereas interobserver variability was <5% in 70–80% of the cases. The remaining cases were examined jointly and consensus was reached. A Histo-score (H-score) based on the percentage of stained neoplastic cells (labelling index-LI) multiplied by staining intensity was calculated, as previously described^45^.

### DNA extraction from paraffin embedded tissues

Sections 10 μm thick were cut from available paraffin-embedded tissue blocks after evaluation of tumour infiltration using the Light Microscope. DNA was extracted from the specimen following a standard DNA extraction kit protocol (Qiamp Micro, Qiagen). The extracted DNA was quantitated on a Picodrop Microliter spectrophotometer.

### Cell culture

BONNA-12 (Leibniz Institute DSMZ German Collection of Microorganisms and Cell Cultures) was cultured in RPMI 1640 (Invitrogen, CA) with 10% heat-inactivated fetal bovine serum (FBS) and 1% penicillin/streptomycin at 37 °C in a humidified 5% CO_2_ atmosphere. Cells were treated with rapamycin (Calbiochem, Darmstadt, Germany) for 48 h at a concentration 500 nm. All experiments were performed in triplicate.

### Stable transfection of BONNA-12 cells

The pBabe-Puro-BRAF-V600E plasmid carrying the *BRAFV600E*was a gift from William Hahn (Addgene plasmid # 15269)^46^. BONNA-12 cells were transfected with the pBabe-Puro-BRAF-V600E plasmid or its empty vector using the Nucleofector solution “V”, the G026 program and a protocol as recommended by Amaxa Biosystems (Lonza, Cologne, Germany). Selection of the clones bearing the *V600E* mutation was performed using puromycin at a concentration 2μg/ml. The *BRAFV600E* mutation in the transfected cells was detected using the Cobas system (Roche Diagnostics, GmbH, Germany). Expression of the *BRAFV600E* mutant plasmid in BONNA-12 was confirmed by Western blot (Fig. 3C).

### Western blot analysis

Protein extraction from 7 BM aspirates with >60% infiltration by HCL as well as BONNA-12 cells was performed using ice-cold RIPA lysis buffer (#9801, Cell Signalling Technology) containing protease and phosphatase inhibitors (Sigma Aldrich). Proteins were separated by SDS-polyacrylamide gel electrophoresis, transferred into nitrocellulose membrane (Porablot NCP, Macherey-Nagel) and incubated with the primary antibodies listed in Supplementary Information Table S5. Goat anti-rabbit IgG-HRP (12–348, Millipore) and goat anti-mouse IgG-HRP (12–349, Millipore) diluted 1:2000 (1hr, room temperature). Immunoreactive bands detection was performed with the SuperSignal WestPico Chemiluminescent HRP Substrate kit (Thermo Scientific). Relative protein amounts were evaluated by densitometric analysis using Image J software (La Jolla, CA, USA) and normalized to the corresponding β-Actin levels. All experiments have been performed at least 3 times.

### MTS proliferation assay

The assessment of BONNA-12 proliferation in the presence or absence of *BRAF*V*600E* and with or without rapamycin treatment was performed with the 3-(4,5-dimethylthiazol-2-yl)-5-(3-carboxymethoxyphenyl)-2-(4-sulfophenyl)-2H-tetrazolium (MTS) assay (Promega Corporation, Fitchburg, WI, USA). Cells were seeded in triplicates in 96-well plates (10^5^ cells/well) and incubated overnight at 37 °C in 5% CO_2_ atmosphere. The next day rapamycin (500 nM, 48 h) was added to cells, followed by addition of MTS (20 μL/well, 4 h). The absorbance was subsequently measured at 490 nm using a microplate reader. Each experiment was conducted in triplicate.

### High Resolution Melting Analysis (HRMA) and Sequencing

*B-RAF* gene exon 15 was screened for mutations in 35 specimens with suitable material, using HRMA on a Light Cycler 480 (Roche Diagnostics, GmbH, Germany), as described elsewhere^47^ and sequenced in order to identify the mutations on an ABI Prism 310 Genetic Analyzer (Applied Biosystems) and/or the Q24 pyrosequencer (QiagenGmblt, Hilden, Germany).

A dilution series of 0%, 5%, 10%, 25%, 50%, 75% and 100% HT29 DNA (*BRAF* mutation) was prepared to validate the sensitivity of the HRMA assay.

### Statistical analysis

p-mTOR, p-4E-BP1, p-p70S6K, p-AKT, p-ERK1/2, BRAFV600E and c-Caspase-3 H-scores, as well as microvascular parameters were statistically analysed as continuous variables. Correlations among the H-scores of these proteins with clinicopathological parameters were tested using non parametric tests (Kruskal-Wallis ANOVA, Mann-Whitney U-test, Spearman tests). Survival analysis was performed using TNT as an endpoint. The effect of various parameters was assessed by using the log-rank test. Numerical variables were categorized on the basis of cut-off values provided by Receiver Operating Characteristics (ROC) analysis. Multivariate analysis was performed using stepwise Cox’s model with the forward strategy. Due to the small number of events in our cohort, in order to ensure power analysis, only those parameters for which there were not any significant missing data and presented with a statistically significant result in univariate analysis were introduced in the multivariate model. Statistical calculations were performed using the Statistical package STATA 11.0 for Windows (Stata Corp. College Station, TX, USA).

## Additional Information

**How to cite this article**: Lakiotaki, E. *et al.* Potential role of AKT/mTOR signalling proteins in hairy cell leukaemia: association with BRAF/ERK activation and clinical outcome. *Sci. Rep.* **6**, 21252; doi: 10.1038/srep21252 (2016).

## Supplementary Material

Supplementary Information

## Figures and Tables

**Table 1:**
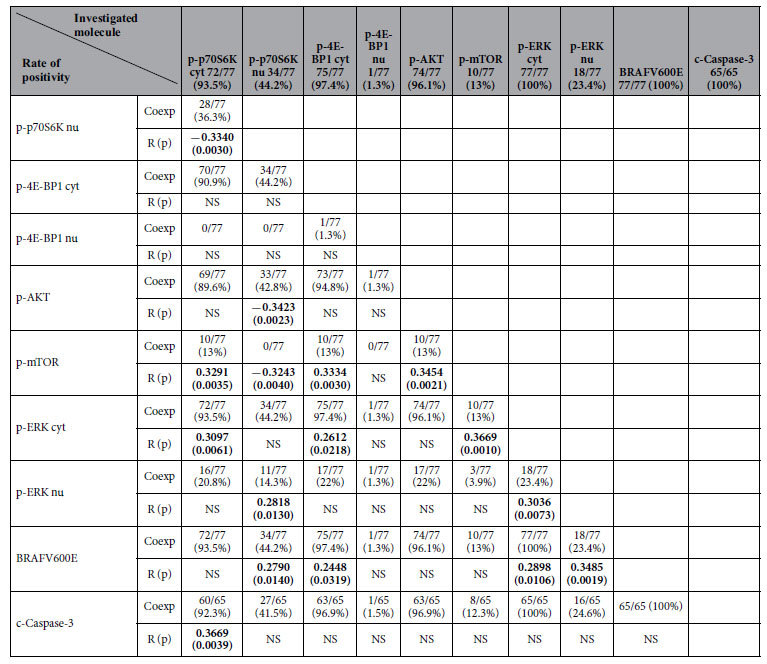
Interrelations and rates of expression/coexpression among the investigated molecules.

Results of Spearman correlation coefficient test. Bold signifies statistical significance. (R: correlation coefficient, p: p-value, Coexp: Coexpression, NS: Not Statistically Significant Correlation). The numbers beneath each investigated molecule refer to the number of positive cases within the total number of investigated cases and the percentage to the rate of positivity of this particular molecule.

**Table 2:** Univariate survival analysis.

Variable	P–value
Age (years) (<53.5 *vs* ≥53.5)	0.0225
Gender	p > 0.10
Splenomegaly (*Absence vs presence*)	p > 0.10
Hepatomegaly (*Absence vs presence*)	0.0408
Haemoglobin (<10 mg/dl *vs* ≥10 mg/dl)	0.0357
Absolute WBC (<3000/μL *vs* ≥3000/μL)	>0.10
Absolute WBC (<1000/μL *vs* ≥1000/μL)	>0.10
Absolute number of circulating HC (<1000/μL *vs* ≥1000/μL)	0.0865
ESR (*Abnormal vs normal*)	>0.10
LDH (*Abnormal vs normal*)	>0.10
Degree of bone marrow infiltration (<80% *vs* ≥80%)	0.0455
Type of first treatment (*interferon vs other*)	0.0347
p-mTOR H-score (*positive vs negative*)	0.0138
Cytoplasmic p-p70S6K H-score (<200 *vs* ≥200)	>0.10
Nuclear p-p70S6K H-score (*positive vs negative*)	>0.10
Cytoplasmic p-4E-BP1 H-score (<225 *vs* ≥225)	<0.0001
Nuclear p-4E-BP1 H-score (*positive vs negative*)	>0.10
p-AKT H-score (<37.5 *vs* ≥37.5)	0.0481
Cytoplasmic p-ERK1/2 H-score (<300 *vs* ≥300)	0.0230
Nuclear p-ERK1/2 H-score (*positive vs negative*)	>0.10
BRAFV600E H-score (<200 *vs* ≥200)	0.0357
c-Caspase-3 (H-score) (<40 *vs* ≥40)	>0.10
Concurrent p-AKT, p-mTOR, p-4-E-BP1 overexpression	<0.0001

Results of univariate survival analysis according to log-rank test for time to next treatment (TNT). The groups compared in each run are illustrated underneath each variable in Italics.

**Table 3:** Cox’s proportional Hazard estimation of TNT.

l	Variable	HR	P-value	95% Confidence interval of HR	
A	p-AKT/cytoplasmic p-mTOR/cytoplasmic p-4E-BP1 *triple positive phenotype vs all other combinations*	69.713	0.001	5.131	947.208
	Degree of bone marrow infiltration	5.369	0.027	1.213	23.763
B	p-4E-BP1 H-score	8.184	0.011	1.631	41.074
	First line treatment *IFN vs non IFN*	0.146	0.035	0.024	0.877
	Degree of bone marrow infiltration	3.787	0.036	1.090	13.155

Cox’s proportional Hazard estimation of TNT including all molecules under study in the entire cohort. Model A is a multivariate model including combination of p-AKT/cytoplasmic p-mTOR/cytoplasmic p-4E-BP1 whereas in Model B p-AKT, p-mTOR and p-4E-BP1 were entered as separate variables. The remaining parameters that were included in both models were the same (those parameters for which there were no significant missing data and presented with a statistically significant result in univariate analysis).
