# Supplementary material for: Potential role of AKT/mTOR signalling proteins in hairy cell leukaemia: association with BRAF/ERK activation and clinical outcome
Source: Sci Rep. 2016 Feb 19;6:21252. doi: 10.1038/srep21252 (PMC4759548; doi:10.1038/srep21252)
Supplement: Supplementary Information [file srep21252-s1.pdf]

Potential role of AKT/mTOR signalling proteins in hairy cell leukaemia: association with BRAF/ERK activation and clinical outcome

Eleftheria Lakiotaki[1¥*], Georgia Levidou[1¥], Maria K. Angelopoulou[2¥], Christos Adamopoulos[3¥], Gerassimos Pangalis[4], George Rassidakis[1,5], Theodoros Vassilakopoulos[2], Gabriella Gainaru[2], Pagona Flevari[2], Sotirios Sachanas[4], Angelica A. Saetta[1], Athanasia Sepsa[1], Maria Moschogiannis[4], Christina Kalpadakis[6], Nikolaos Tsesmetzis[5], Vassilios Milionis[1], Ilenia Chatziandreou[1], Irene Thymara[1], Panayiotis Panayiotidis[7], Maria Dimopoulou[2], Eleni Plata[2], Konstantinos Konstantopoulos[2], Efstratios Patsouris[1], Christina Piperi[3#], Penelope Korkolopoulou[1#]

1. Department of Pathology, University of Athens, Medical School, Greece; 2. Department of Haematology and Bone Marrow Transplantation, University of Athens, Medical School, Greece; 3. Department of Biological Chemistry, University of Athens, Medical School, Greece; 4. Department of Haematology, Athens Medical Centre, Psychikon Branch, Greece; 5. Department of Oncology-Pathology, Cancer Centrum Karolinska, Karolinska Institutet, Stockholm, Sweden; 6. Department of Haematology, University of Crete, Heraklion, Greece; 7. 1st Department of Propaedeutic Internal Medicine, University of Athens, Medical School, Greece

[¥]Equal contribution

Senior authors

Table S1**: Associations between BRAFV600E, cytoplasmic p-ERK, nuclear p-ERK, p-AKT, p-mTOR, p-p70S6K and p-4E-BP1, clinicopathological features and microvascular characteristics.** Bold signifies statistical significance. (R: correlation coefficient, p: p-value, NS: Not Statistically Significant Correlation)

H-scores / ***Variables***		**BRAFV 600E**	**p-ERK cyt**	**p-ERK nu**	**p-AKT**	**p-mTOR**	**p-p70S6K**	**p-4E-BP1**
Age	**R**							
	**p**	NS	NS	NS	NS	NS	NS	NS
Gender	**p**	NS	NS	NS	NS	NS	NS	NS
Splenomegaly *(Absence vs presence)*	**p**	NS	NS	NS	**0.0591***	NS	NS	NS
Hepatomegaly *(Absence vs presence)*	**p**	NS	NS	NS	NS	NS	NS	NS
LDH *(Abnormal vs normal)*	**p**	NS	NS	NS	NS	NS	NS	NS
Lymphadenopathy *(Absence vs presence)*	**p**	NS	NS	NS	NS	NS	NS	NS
Gamma globulins *(Abnormal vs normal)*	**p**	NS	NS	NS	NS	NS	NS	NS
Haemoglobin *(<10mg/dl vs ≥10 mg/dl)*	**p**	NS	**0.0215**	NS	NS	NS	NS	NS
Absolute White Blood Count	**R**							
	**p**	NS	NS	NS	NS	NS	NS	NS
Absolute Neutrophil Count	**R**	**-0.2509**						
	**p**	**0.0531**	NS	NS	NS	NS	NS	NS
Absolute number of circulating Hairy Cells	**R**						**(nuclear) -0.429**	
	**p**	NS	NS	NS	NS	NS	**0.0100**	NS
	**R**	**-0.2492**	**-0.2835**		**0.2715**			

Absolute Platelet Counts	**p**	**0.0471**	**0.0232**	NS	**0.0030**	NS	NS	NS
BM infiltration at diagnosis	**R**	NS	NS	NS	NS	NS	NS	NS
	**p**							
HC Index	**R**							
	**p**	NS	NS	NS	NS	NS	NS	NS
Microvessel Density	**R**	NS	NS	NS	NS	NS	NS	NS
	**p**							
Major Axis Microvessel Length	**R**	NS	NS	NS	NS	NS	NS	NS
	**p**							
Minor Axis Microvessel Length	**R**	NS	NS	NS	NS	NS	NS	NS
	**p**							
Microvessel Area	**R**	NS	NS	NS	NS	NS	NS	NS
	**p**							
Microvessel Perimeter	**R**	NS	NS	NS	NS	NS	NS	NS
	**p**							
Microvessel Shape factor	**R**	NS	NS	**0.3598**	NS	NS	NS	NS
	**p**			**0.0338**				
Microvessel Compactness	**R**	NS	NS	NS	NS	NS	NS	NS
	**p**							
Microvessel Feret Diameter	**R**	NS	NS	NS	NS	NS	NS	NS
	**p**							
Microvessel Branching	**R**	NS	NS	NS	NS	NS	NS	NS
	**p**							

*marginal statistical significance

Table S2*: *AKT/mTOR pathway components and p-ERK1/2 expression in normal BM

Protein	Expression pattern
**p-ERK**	only in endothelial cells
**p-AKT**	Faint staining in precursor erythroid cells and a small number of megakaryocytes
**p-mTOR**	nuclear positivity in a limited number of megakaryocytes, faint cytoplasmic positivity in plasma cells
**p-4E-BP1**	Faint nuclear immunopositivity in myeloid cells and megakaryocytes
**p-p70S6K**	Moderate nuclear staining in myeloid cells and megakaryocytes, erythroid precursor unstained

***Table S3*: AKT/mTOR pathway components and p-ERK1/2 expression in other B cell lymphomas**

**B cell lymphoma type**	**p-ERK1/2 positive cases / Total number of cases**	**Other comments related to p-ERK1/2 expression**
MCL	0/5	
CLL	0/5	
MZL	0/5	
HCLv	0/1	
**B cell lymphoma type**	**p-AKT positive cases / Total number of cases**	**Other comments related to p-AKT expression**
MCL	4/5	Expression in 2-15% of cells
CLL	5/5	Faint expression in proliferation centers moderate expression in paraimmunoblasts
MZL	1/5	Moderate expression (5%) limited to blast cells
HCLv	1/1	Faint cytoplasmic expression (20%)
**B cell lymphoma type**	**p-mTOR positive cases / Total number of cases**	**Other comments related to p-mTOR expression**
MCL	5/5	Moderate and strong cytoplasmic and nuclear immunostaining (40-95%)
CLL	5/5	Faint expression (25-90%), more pronounced in the proliferation centers

MZL	4/5	Faint cytoplasmic expression, range 60-80%
HCLv	1/1	Faint cytoplasmic expression (80%)
**B cell lymphoma type**	**p-4E-BP1 positive cases / Total number of cases**	**Other comments related to p-4E-BP1 expression**
MCL	5/5	Faint to moderate positivity, range 5%-70%
CLL	3/5	Faint to moderate nuclear and cytoplasmic positivity, mainly in proliferation centers, range 10-70%
MZL	3/5	Weak expression in 5-20% of cells
HCLv	1/1	Faint cytoplasmic expression in 95% of cells
**B cell lymphoma type**	**p-p70S6K positive cases / Total number of cases**	**Other comments related to p-p70S6K expression**
MCL	5/5	Moderate to strong nuclear expression, range (40-90%)
CLL	5/5	Moderate nuclear expression, mainly in the proliferation centers, range (50-90%)
MZL	5/5	Faint to moderate nuclear expression, range (55-95%)
HCLv	1/1	Weak cytoplasmic expression in 95% of cells

***Table S4*: Clinicopathologic Characteristics, Survival and Treatment Information of the 77 HCL patients**

**Characteristic**	**Patients**
Males / Females	78 / 22
Splenomegaly	43%
Hepatomegaly	19.4%
Lymphadenopathy	5%
Elevated LDH	10.8%
Abnormal gamma globulins	35.2%

**Characteristic**	**Median**	**Range**
Age (years)	53.5	(30-88)
Haemoglobin (g/dL)	11.4	(3.8-15.8)
WBC (X10^9/L)	3.1	(1-54)
ANC (X10^9/L)	0.52	(0.03-6.248)
HC (X10^9/L)	1.054	(0-48.6)
PLT (X10^9/L)	81	(18-264)
BM infiltration at diagnosis (%)	80	10-100
HC Index	0.5625	(0.045-1.0)

**Flow Cytometry Findings**	**% of Patients**
Positive Antigen Expression	
Pan B cell antigens (n*=77)	100
CD79b (n*= 42)	97.6
Kappa (n*=77)	50
Lambda (n*=77)	50
CD10 (n*= 38)	23.7
CD23 (n*= 39)	38.5†

CD38 (n*= 34)	29.4†
CD11c (n*= 45)	95.6
CD25 (n*= 45)	97.8
CD103 (n*= 44)	97.7
FMC7 (n*= 44)	100
Annexin A1(n=77)	100

**Survival and Treatment Information**	**Range**
Median follow-up	136.2 months (range 2-393)
10-year overall survival	88.4%
Disease specific survival	98.5%
First treatment	
IFN-α	82.2%
Purine analogues	13.7%
Splenectomy	1 patient
G-CSF/rhEPO	1 patient
No treatment	1 patient
Next treatment	30/72 patients
Median TNT	177 months (range 133.8-221)
IFN-α	12 patients
Purine analogues	6 patients
Other**	12 patients

*LDH: lactate dehydrogenase, WBC: white blood cells, ANC: absolute neutrophil counts, HC: absolute hairy cell counts, PLT: platelet counts, BM: bone marrow, G-CSF/rhEPO: granulocyte-colony stimulating factor/ recombinant human erythropoietin *: n designates the number of cases in which the corresponding antigen was studied, **other: rituximab, G-CSF/rhEPO, steroids, †: CD23 and CD38 were significantly correlated (p= 0.001)*

Table S5: **The characteristics of primary antibodies used in immunohistochemical analysis and Western immunoblotting**

Protein	Clone	Company	Catalog no.	Raised in	Positive controls	Antigen retrieval method	Dilution and incubation time	Western blot (antibody)	Western blot (dilution)
p-mTOR (Ser2448)	Monoclonal	Cell Signaling Technology, USA	#2976	Rabbit	Human breast cancer	Citrate buffer, pH 9	1:50, overnight	#5536, Cell Signaling Technology, USA	1:1000
p-p70S6K (Thr421/Ser424) [specific for p70 subunit]	Polyclonal	Santa Cruz Biotechnology, USA	sc-7984-R	Rabbit	Human colon carcinoma	Citrate buffer, pH 6	1:250, overnight	sc-8416, Santa Cruz Biotechnology, USA	1:200
p-4E-BP1 (Thr37/46) 236B4	Monoclonal	Cell Signaling Technology, USA	#2855	Rabbit	Human breast cancer	Citrate buffer, pH 6	1:800, overnight	sc-101624, Santa Cruz Biotechnology, USA	1:200
AKT-pS473 [phosphorylation site specific]	Polyclonal, Clone 14-5	Dako, Dakopatts	M3628	Rabbit	Mouse kidney	Citrate buffer, pH 9	1:20, 1h	M3628, Dako, Dakopatts	1:200

ERK1/2 [phosphorylated at Tyr 204 and p-ERK2]	Monoclonal	Santa Cruz Biotechnology, USA	sc-7383	Mouse	Human breast cancer	Citrate buffer, pH 9	1:50, overnight	sc-7383, Santa Cruz Biotechnology, USA	1:200
BRAFV600E [clone VE1]	Monoclonal	Spring, Bioscience	E 19290	Mouse	Human colon carcinoma	Citrate buffer, pH 9	1:500, overnight	E 19290, Spring, Bioscience	1:200
c-Caspase-3 (Asp 175)	Monoclonal	Cell Signaling Technology, USA	#836	Rabbit	Burkitt lymphoma	Citrate buffer, pH 9	1:80, 30 min	-	-
Actin [clone C4]	-	-	-	-	-	-	-	MAB1501, Millipore, MA	1:5000

***Figure S1*: p-AKT expression in mantle cell lymphoma. p-mTOR and p-p70S6K expression in chronic lymphocytic leukaemia (X400)**

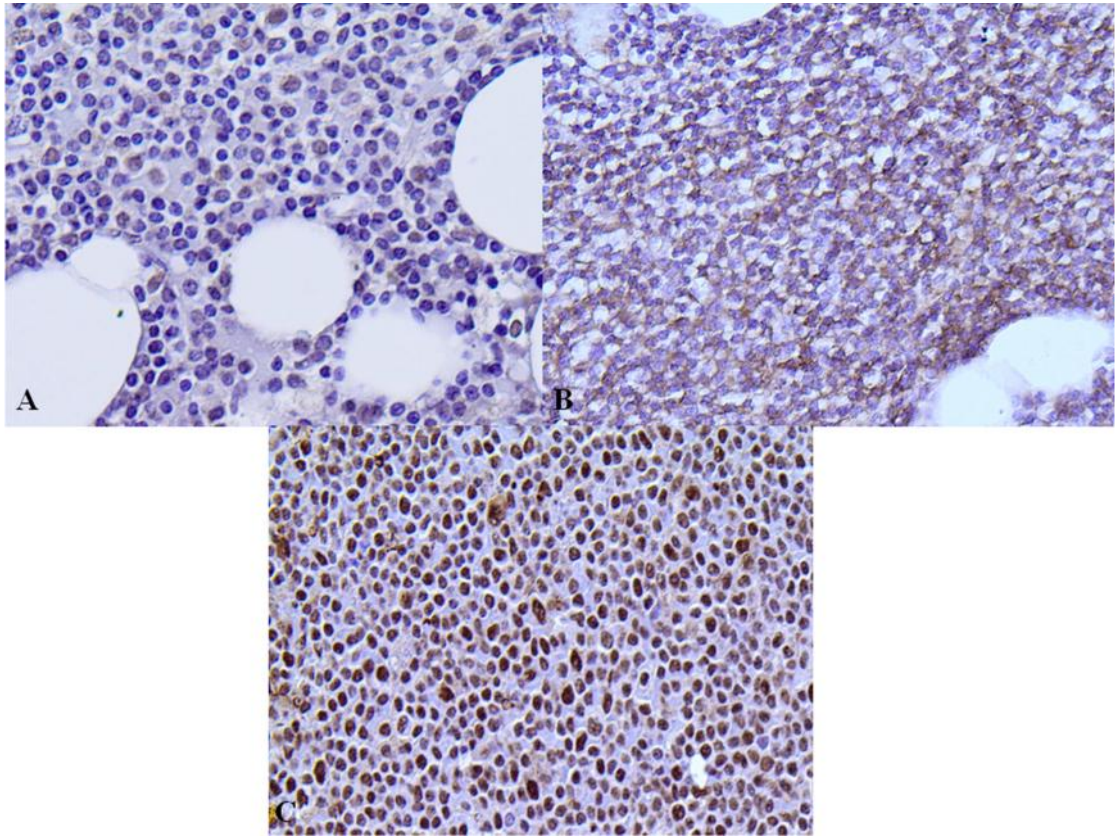

Figure S1 Legend:

(A): Cytoplasmic p-AKT immunoreactivity in mantle cell lymphoma.

(B): Cytoplasmic p-mTOR immunoreactivity in chronic lymphocytic leukaemia. The expression was faint in the majority of cases and more pronounced in the proliferation centers

(C): Nuclear and cytoplasmic p-p70S6K immunoreactivity in chronic lymphocytic leukaemia. The expression was mostly moderate and more pronounced in the proliferation centers

Figure S2**: p-4E-BP1 expression in chronic lymphocytic leukaemia, mantle cell lymphoma and marginal zone lymphoma (X400)**

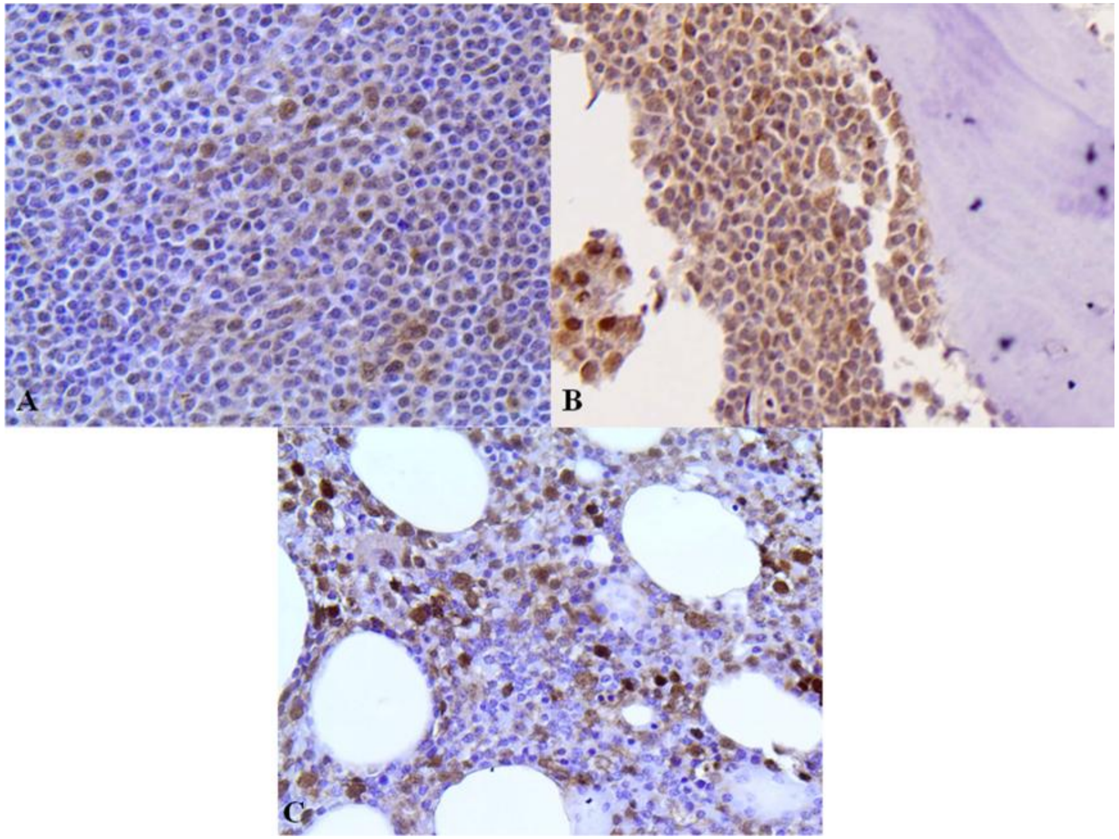

Figure S2 Legend:

(A): Cytoplasmic and nuclear p-4E-BP1 expression in chronic lymphocytic leukaemia. The expression was more pronounced in the proliferation centers

(B): Faint to moderate cytoplasmic and nuclear p-4E-BP1 expression in mantle cell lymphoma

(C): Cytoplasmic and nuclear p-4E-BP1 expression in marginal zone lymphoma
